# Factors associated to quality of life in patients with leprosy

**DOI:** 10.31744/einstein_journal/2021AO5936

**Published:** 2021-08-12

**Authors:** Graziele Ferreira Pinto, Raquel Aparecida Rodrigues Nicácio, Fernanda Rocha Anjos de Oliveira, Isabella Alcantara de Oliveira, Rauni Jandé Roama Alves, Débora Aparecida da Silva Santos, Letícia Silveira Goulart

**Affiliations:** 1 Universidade Federal de Rondonópolis RondonópolisMT Brazil Universidade Federal de Rondonópolis, Rondonópolis, MT, Brazil.

**Keywords:** Leprosy, Quality of life, Epidemiologic factors, Public Health, Epidemiology

## Abstract

**Objective:**

To evaluate quality of life and associated factors in patients with leprosy.

**Methods:**

A cross-sectional study with 63 people diagnosed as leprosy, seen at a reference service for the disease in the southeastern region of Mato Grosso, Brazil. The questionnaire World Health Organization Quality of Life Bref was used to evaluate quality of life. Simple and multiple linear regressions evaluated the association between sociodemographic variables and quality of life domains.

**Results:**

The highest mean of quality of life was observed in the psychological domain (16.28±2.30), and the lowest in the environmental domain (13.86±2.21). Females, individuals with no partners, and people who owned their own house had the lowest quality of life means within the psychological domain. People who did not receive visits by Community Health Workers had the lowest means in quality of life within the environmental domain. Multivariate analysis revealed that the best quality of life was associated to self-reported white skin color within the environmental domain, and the worst quality of life was associated to less schooling within the physical domain.

**Conclusion:**

This study showed the influence of sociodemographic factors on the quality of life of patients with leprosy, and indicated the need for comprehensive health care, considering the social determinants of health.

## INTRODUCTION

Leprosy is a compulsory reporting infectious disease, whose etiologic agent is the bacillus *Mycobacterium leprae.* ^( 1 )^ Characterized as a chronic and millennial disease, it is still considered a serious public health problem today, with more than 200 thousand new cases reported annually worldwide. Brazil ranks second in number of cases: 25,218 new cases in 2016.^( 2 )^

Mato Grosso is the Brazilian state that ranks first in leprosy detection coefficient. In 2016, 2,681 new cases were recorded, totaling up 80.16/100 thousand inhabitants. In the city of Rondonópolis (MT), 102 new cases were reported in 2016, resulting in 45.88/100 thousand inhabitants, and the municipality and the state were characterized as hyperendemic regions for the disease.^( 3 , 4 )^

According to the World Health Organization (WHO), quality of life (QoL) is defined as “an individual’s perception of their position in life, in the context of culture and value systems in which they live and in relation to their goals, expectations, standards, and concerns”.^( 5 )^ Its understanding has surpassed the concept of being synonymous with health and well-being, as it involves health, education, transportation, housing, work, and participation in decisions concerning them.^( 6 )^

Leprosy is capable of negatively affecting the QoL of individuals. In general, the disease brings much physical discomfort and pain to the body, preventing the individual from working and performing daily tasks, limiting their actions, and restricting leisure activities.^( 7 )^

Prejudice and stigmas imposed by society due to dermatological alterations and physical disabilities contribute to the emergence of feelings, such as low self-esteem, shame, rejection, and isolation in the family, social, academic, and professional environments. In addition, prolonged treatment and its adverse effects also contribute negatively to the QoL of leprosy patients.^( 2 , 8 )^

In the field of health, QoL has a value attributed to life based on functional deteriorations, perceptions, and social conditions induced by diseases, injuries, and treatments. Its study and assessment are intended to humanize care.^( 9 )^ In this context, a better understanding of the QoL of leprosy patients can contribute to the expansion of comprehensive care, as well as to the development of interventions that can provide improvement in the service delivered to this population.

## OBJECTIVE

To evaluate quality of life and its associated factors in leprosy patients.

## METHODS

This is a descriptive study with a cross-sectional design and quantitative approach. All patients diagnosed with leprosy, aged 18 years or older, who attended the *Serviço de Atendimento Especializado* Abel Christian Rodrigues de Melo, in the city of Rondonópolis (MT), during the month of September 2019 were enrolled in the research. There were three refusals, totaling up 63 patients.

Sociodemographic data were collected using a previously tested structured questionnaire. The World Health Organization Quality of Life Bref (WHOQOL-BREF)^( 10 )^ questionnaire was used in its Portuguese version, available at https://www.ufrgs.br/qualidep/qualidade-de-vida/projeto-whoqol-bref/50-whoqol-bref. Validated by the WHO, it consists of 26 items, two of which are self-assessments of health, and 24 referring to four domains: physical, psychological, social relationships, and environment.

The dependent variable was QoL, and the independent variables were age, sex, self-reported skin color, schooling, income, marital status, home ownership, number of rooms in the house, number of people living in the same house, health insurance affiliation, sanitation, use of Family Health Strategy (FHS) services and home visits by Community Health Workers (CHW).

Data from the WHOQOL-BREF questionnaire were initially analyzed in Excel, as described by Pedroso et al., using the platform available at http://www.brunopedroso.com.br/whoqol-bref.html.^( 11 )^ In the WHOQOL-BREF questions, higher means suggest a better perception of QoL.

The variables that presented with p<0.20 in the univariate analysis were inserted into the multiple linear regression model. The significance level adopted was p≤0.05. For the statistical analyses, Office 365 Excel and IBM (SPSS) 26.0 for Windows were used.

During the research, ethical precepts were followed according to Resolution 466 of December 12, 2012, from the National Health Council of the Ministry of Health, for research with human beings.^( 12 )^ This study was part of the survey entitled Leprosy: Analysis of Cases and Program Management in a Hyperendemic Municipality. It was approved by the Research Ethics Committee of the *Universidade Federal de Rondonópolis* (UFR), opinion 3.036.673, and CAAE: 97441618.2.0000.8088. All study participants signed the Informed Consent Form (ICF).

## RESULTS

The mean age of those surveyed was 51.74 years. Most of the participants were men (63.50%; n=40), aged from 30 to 50 years (44.40%; n=28), self-declared as brown-skinned (58.70%; n=37), who had a partner (62.90%; n=39), had an income higher than two monthly minimum wages (54.0%, n=34), had studied up to 8 years (69.80%; n=44), had a sewage system (60.30%; n=38), did not own their own house (75.80%; n=47), had more than four rooms in their house (43.50%; n=27), three or more people lived in their residence (55.60%; n=35), who received home visits from the CHW (60.30%; n=38), used the FHS (84.10%; n=53), and were not affiliated to a health insurance plan (81.0%; n=51).

**Figure 1:**
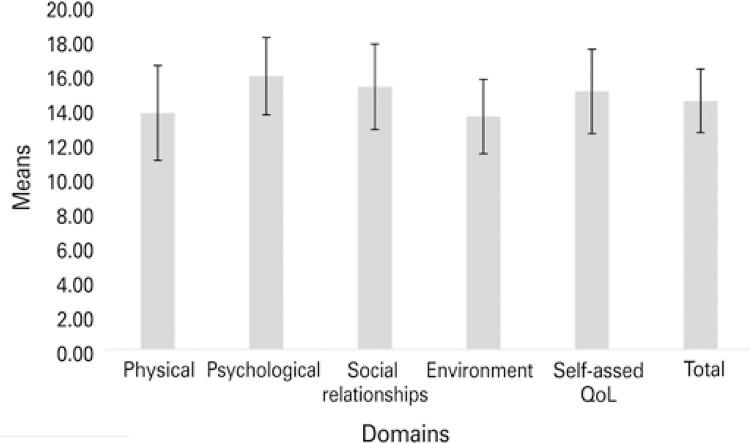
Centrality and dispersion measures of scores in the quality of life domains of the World Health Organization Quality of Life Bref, from individuals with leprosy followed at a reference service

Figure 1 shows the mean scores of the QoL domains in the WHOQOL-BREF of the study population. The mean WHOQOL-BREF score in overall QoL was 14.80±1.89. The highest mean was seen in the psychological domain (16.28±2.30), and the lowest, in the environment domain (13.86±2.21).

QoL: quality of life.

Women (p=0.036), individuals without a partner (p=0.016), and those who owned their own home (p=0.033) showed lower QoL values in the psychological domain ( Tables 1 and 2 ).

**Table 1:** Distribution of mean overall quality of life scores and in each domain of the World Health Organization Quality of Life Bref for the leprosy patients seen at a reference service, according to their intraindividual characteristics

Variables	Mean (SD) of the WHOQOL-BREF scores				

	Physical	Psychological	Social Relationships	Environment	Overall QoL
Sex					
Female	13.46 (2.90)	15.48 (2.65)	15.83 (2.25)	13.39 (2.12)	14.28 (1.95)
Male	14.43 (2.76)	16.73 (1.97)	15.53 (2.73)	14.13 (2.23)	15.09 (1.82)
p value	0.196	0.036	0.664	0.202	0.101
Age, years					
30-50	14.18 (3.26)	16.09 (2.19)	15.76 (2.46)	13.52 (2.23)	14.71 (2.02)
51-59	13.53 (2.86)	16.24 (2.79)	15.44 (2.68)	14.42 (2.53)	14.80 (2.18)
> 60	14.53 (1.85)	16.62 (1.94)	15.66 (2.69)	13.80 (1.68)	14.94 (1.31)
p value	0.567	0.768	0.914	0.390	0.932
Self-declared skin color					
White	13.75 (3.39)	16.27 (3.18)	15.37 (3.23)	14.49 (2.26)	14.83 (2.26)
Brown	14.35 (2.47)	16.16 (2.01)	15.75 (2.35)	13.94 (2.09)	14.92 (1.73)
Black	13.59 (3.25)	16.74 (1.50)	15.70 (2.08)	12.33 (2.09)	14.24 (1.88)
p value	0.066	0.801	0.882	0.054	0.633
Marital status					
With no partner	13.79 (2.74)	15.39 (2.50)	15.13 (2.62)	13.67 (2.16)	14.41 (1.85)
With partner	14.39 (2.78)	16.84 (2.03)	16.00 (2.48)	14.08 (2.16)	15.12 (1.81)
p value	0.413	0.016	0.197	0.473	0.142

SD: standard deviation; WHOQOL-BREF: World Health Organization Quality of Life Bref; QoL: quality of life.

**Table 2:** Distribution of mean overall quality of life scores and in each domain of the World Health Organization Quality of Life Bref for leprosy patients seen at a reference service, according to their socioeconomic characteristics

Variables	Mean (SD) of the WHOQOL-BREF scores				

	Physical	Psychological	Social relationships	Environment	Overall QoL
Schooling					
Up to 8 years	13.71 (3.00)	16.34 (2.33)	15.70 (2.65)	13.69 (2.39)	14.66 (2.06)
More than 8 years	14.92 (2.24)	16.10 (2.28)	15.51 (2.36)	14.26 (1.68)	15.12 (1.43)
p value	0.122	0.704	0.790	0.347	0.383
Income					
< 1 minimum monthly wage	13.08 (3.17)	15.70 (2.55)	15.17 (2.65)	13.29 (2.35)	14.14 (2.10)
> 2 minimum monthly wages	14.92 (2.20)	16.76 (1.97)	16.04 (2.42)	14.34 (1.98)	15.35 (1.52)
p value	0.009	0.067	0.180	0.058	0.011
Home ownership					
Yes	13.40 (3.23)	15.20 (1.97)	15.47 (2.56)	13.23 (2.49)	14.19 (1.97)
No	14.40 (2.58)	16.65 (2.32)	15.74 (2.57)	14.15 (2.01)	15.07 (1.77)
p value	0.226	0.033	0.717	0.150	0.111
Number of rooms					
1-3	12.95 (2.87)	14.81 (3.43)	15.11 (3.26)	13.50 (2.16)	13.91 (2.05)
4	14.72 (2.43)	16.82 (1.69)	16.46 (1.69)	14.26 (2.47)	15.35 (1.88)
> 5	14.03 (2.97)	16.30 (2.68)	15.11 (2.09)	13.76 (1.83)	14.70 (1.65)
p value	0.241	0.079	0.120	0.572	0.112
Number of people in the home					
< 2	13.73 (2.40)	15.73 (1.69)	15.46 (2.85)	13.68 (2.32)	14.50 (1.60)
> 3	14.50 (3.00)	16.74 (2.63)	15.85 (2.33)	14.13 (2.02)	15.13 (1.99)
Valor de p	0.285	0.087	0.554	0.417	0.189
Sewage system					
Yes	14.39 (2.56)	16.40 (2.34)	15.61 (2.50)	13.99 (1.92)	14.92 (1.68)
No	13.60 (3.18)	16.08 (2.28)	15.68 (2.28)	15.68 (2.67)	14.61 (2.20)
p value	0.281	0.589	0.921	0.583	0.522

SD: standard deviation; WHOQOL-BREF: World Health Organization Quality of Life Bref; QoL: quality of life.

Individuals with lower income displayed lower mean scores in the overall QoL (p=0.011) and in the physical domain (p=0.009) ( Table 2 ).

Patients who did not receive a visit from a CHW showed worse QoL in the environment domain (p=0.040) ( Table 3 ).

**Table 3:** Distribution of mean overall quality of life scores and in each domain of the World Health Organization Quality of Life Bref for leprosy patients seen at a reference service, according to their characteristics of access healthcare services

Variables	Mean (SD) of the WHOQOL-BREF scores				

	Physical	Psychological	Social relationships	Environment	Overall QoL
Utiliza FHS					
Yes	13.98 (2.71)	16.26 (2.21)	15.90 (2.52)	13.80 (2.22)	14.78 (1.84)
No	14.57 (3.50)	16.33 (2.85)	14.27 (2.35)	14.20 (2.21)	14.91 (2.24)
p value	0.551	0.931	0.063	0.601	0.843
Receive visits from CHW					
Yes	13.95 (3.07)	16.31 (2.43)	15.86 (2.46)	14.32 (2.23)	14.95 (2.03)
No	14.26 (2.46)	16.21 (2.12)	15.31 (2.70)	13.16 (2.01)	14.57 (1.68)
p value	0.676	0.864	0.404	0.040	0.448
Health insurance plan					
Yes	13.24 (2.78)	15.94 (2.40)	15.66 (2.28)	13.79 (2.05)	14.42 (1.59)
No	14.27 (2.83)	16.35 (2.29)	15.63 (2.63)	13.88 (2.26)	14.89 (1.96)
p value	0.257	0.584	0.969	0.904	0.451

SD: standard deviation; WHOQOL-BREF: World Health Organization Quality of Life Bref; QoL: quality of life; FHS: Family Health Strategy; CHW: Community Health Worker.

The results of the multivariate regression are shown on table 4 . After the adjusted analysis, the variables that remained associated with QoL were schooling and self-reported skin color. A lower schooling level was associated with lower QoL in relation to the physical domain, and being white was associated with better QoL in the environment domain.

**Table 4:** Multivariate analysis of mean overall quality of life scores in each domain of the World Health Organization Quality of Life Bref for leprosy patients seen at a reference service

Variables	Correlation coefficient				

	Physical (95%CI)	Psychological (95%CI)	Social relationships (95%CI)	Environment (95%CI)	Overall QoL (95%CI)
Sex	-0.50 (-2.16 - -1.17)	-0.27 (-1.60-1.06)	*	*	-0.27 (-1.51-0.96)
Self-declared skin color	*	*	*	1.39^†^ (0.28-2.50)	*
Schooling	-1.78^†^ (-3.51 - -0.04)	*	*	*	*
Income	*	-0.016 (-0.67-0.64)	0.39 (-0.30-1.08)	-0.18 (-0.85-0.49)	0.08 (-0.52-0.67)
Marital status	*	-0.40 (-1.72-0.92)	*	*	0.10 (-1.23-1.03)
Home ownership	*	0.98 (-0.47-2.43)	*	0.53 (-1.12-2.19)	0.35 (-0.90-1.60)
Number of rooms	*	-0.313 (-1.55-0.93)	0.63 (-0.71-1.97)	*	-0.06 (-1.13-1.01)
Number of people in the home	*	-0.363 (-1.63-0.90)	*	*	-0.59 (-1.67-0.50)
Use FHS	*	*	0.27 (-1.59-2.13)	*	*
Receive visits from CHW	*	*	*	0.61 (-0.75-1.97)	*

* p>0.20 in the univariate analysis, not informed in the logistic regression. Multivariate analysis; ^†^ p≤0.05.

95%CI: 95% confidence interval; QoL: quality of life; FHS: Family Health Strategy; CHW: Community Health Worker.

## DISCUSSION

As a disease that has been reaching large proportions worldwide, leprosy can affect the individual in several dimensions and impact on their QoL. In this study, most participants were men, self-declared as brown, living with a partner, with family income equal to or higher than two minimum monthly wages, and reporting up to 8 years of schooling. These data corroborate previous research, which indicate that men, people with poor socioeconomic conditions, those with low schooling levels, and individuals with low income are at a higher risk of acquiring the disease.^( 13 - 16 )^

In this study, we demonstrated the WHOQOL-BREF domain with the lowest mean was the environment domain. This domain refers to protective facets, such as availability and quality of health and social care, and the opportunity to acquire new information and skills.^( 17 )^These factors may interfere in the process of self-care and therapeutic success in leprosy. In this sense, it is important that environment parameters be considered for the adoption of health promotion strategies in this population group.

Similarly, a survey with leprosy patients monitored in specialized reference centers in the city of Sergipe (AL) also described the environment domain showed the lowest scores in the WHOQOL-BREF.^( 18 )^ A study carried out with patients from leprosy self-care support groups in the city of Recife (PE) and its metropolitan region found lower QoL scores for the environment and physical domains, both with the same mean.^( 19 )^ However, previous studies observed the physical domain showed the lowest mean QoL scores in populations of individuals with leprosy.^( 16 , 20 )^

Despite the fact the WHOQOL-BREF allows comparing its results among different populations, one must take into consideration the notion of QoL suffers personal influences related to the individual’s ability to deal with certain situations. This fact, added to local environment, social, and cultural factors, may influence the test results when performed in different locations.^( 21 )^

In this study, the factors that remained associated with QoL in multiple regressions were self-reported skin color and schooling. Brouwers et al., showed that maintaining a family, health satisfaction, activity limitations, and type of house were factors significantly related to worse QoL in leprosy patients in Nepal.^( 22 )^ Tsutsumi et al., found the factors that potentially contribute to worse QoL in leprosy patients were the presence of perceived stigma, fewer years of study, presence of deformities, and a lower annual income.^( 16 )^

After multivariate analysis, it was found that the best QoL was associated with the self-declaration of white skin color in the environment domain. Data from the Brazilian Institute of Geography and Statistics (IBGE - *Instituto Brasileiro de Geografia e Estatística* ) indicate the black or brown population is disadvantaged in some issues, such as labor market, average monthly income, basic sanitation, Internet access, years of study, and public security.^( 23 )^ These issues are encompassed in the environment domain, which may justify the fact that white patients have a better QoL. This result reinforces the need to adopt public policies that seek to reduce social inequalities, ensuring better living conditions for all segments of the population.

Lower schooling levels was a factor negatively associated with QoL in the physical domain, and a similar result was verified in leprosy patients seen at a hospital in Bangladesh.^( 16 )^ Leite et al., analyzed the QoL of individuals with leprosy in the cities of Bayeux (PA) and Natal (RN). The authors found that the lowest means of overall QoL and social relationships domain were observed in individuals with a lower level of schooling.^( 20 )^

The physical domain of the WHOQOL-BREF aims to assess to what extent energy and fatigue, pain and discomfort, sleep and rest are affected.^( 16 )^A low educational level can cause difficulties in understanding the orientations given by the FHS, as well as in the therapeutic follow-up.^( 21 )^In this context, individuals with less education may have more difficulties in self-care, which can impact their QoL. This finding is an important indicator to be observed by managers and professionals when planning health actions, to refine the view and propose strategies directed towards less educated patients.^( 21 )^

Currently, few Brazilian studies assess the factors associated with QoL of patients with leprosy, demonstrating the importance of expanding studies addressing this topic. As for limitations of this study, we can mention the data collection method, in which the information is self-reported, regarding both sociodemographic data and the WHOQOL-BREF, which may be subject to recall bias. The WHOQOL-BREF, however, is a validated and widely used instrument. Another limitation refers to the sample studied, which involved a small group of patients followed up by a reference service, with no sample of patients from the city. Further more comprehensive studies should be carried out.

## CONCLUSION

Individuals with leprosy followed up at a reference service showed low quality of life scores. Perceptions of worse quality of life were observed among women, individuals with low levels of schooling, brown- or black-skinned, without a partner, with no home of their own, and who did not receive visits from Community Health Workers. The results indicated the impact of sociodemographic factors on quality of life, reinforcing the need for comprehensive health care, understanding the social determinants of health in this population.
