# Supplementary material for: Factors associated to quality of life in patients with leprosy
Source: Einstein (Sao Paulo). 2021 Aug 12;19:eAO5936. doi: 10.31744/einstein_journal/2021AO5936 (PMC8362908; doi:10.31744/einstein_journal/2021AO5936)
Supplement: Translation

# Translation: Fatores associados à qualidade de vida em pacientes com hanseníase

**Authors:** Graziele Ferreira Pinto, Raquel Aparecida Rodrigues Nicácio, Fernanda Rocha Anjos de Oliveira, Isabella Alcantara de Oliveira, Rauni Jandé Roama Alves, Débora Aparecida da Silva Santos, Letícia Silveira Goulart

**Affiliations:** 1 Brasil Universidade Federal de Rondonópolis, Rondonópolis, MT, Brasil.

**Keywords:** Hanseníase, Qualidade de vida, Fatores epidemiológicos, Saúde Pública, Epidemiologia

## Abstract

**Objetivo:**

Avaliar a qualidade de vida e seus fatores associados em pacientes com hanseníase.

**Métodos:**

Estudo transversal com 63 indivíduos com diagnóstico de hanseníase acompanhados em um serviço de atendimento especializado da região sudeste de Mato Grosso. Para avaliação da qualidade de vida, aplicou-se o questionário *World Health Organization Quality of Life Bref* . Utilizou-se regressão linear simples e múltipla para avaliar a associação entre varáveis sociodemográficas e os domínios de qualidade de vida.

**Resultados:**

A maior média de qualidade de vida foi observada no domínio psicológico (16,28±2,30) e a menor, no domínio ambiente (13,86±2,21). As mulheres, indivíduos sem companheiro e aqueles que possuíam casa própria apresentaram menores médias de qualidade de vida no domínio psicológico. Os indivíduos que não receberam visita de Agentes Comunitários de Saúde apresentaram menores médias de qualidade de vida no domínio ambiente. Após análise multivariada, verificou-se que melhor qualidade de vida esteve associada à autodeclaração de cor branca no domínio ambiente, e pior qualidade de vida foi associada à menor escolaridade no domínio físico.

**Conclusão:**

Este estudo evidenciou a influência dos fatores socioedemográficos sobre a qualidade de vida de pacientes com hanseníase, indicando a necessidade de realização de uma assistência em saúde de maneira integral, compreendendo os determinantes sociais da saúde.

## INTRODUÇÃO

A hanseníase é uma doença infectocontagiosa, de notificação compulsória, que tem como agente etiológico o bacilo *Mycobacterium leprae.* ^( 1 )^Caracterizada como uma doença crônica e milenar, é considerada ainda um grave problema de saúde pública na atualidade, com mais de 200 mil novos casos registrados anualmente em todo o mundo, sendo o Brasil o país com segundo maior número de casos: 25.218 novos casos no ano de 2016.^( 2 )^

Mato Grosso é o estado brasileiro que ocupa o primeiro lugar em coeficiente de detecção da hanseníase. Em 2016, foram registrados 2.681 casos novos, somando 80,16/100 mil habitantes. No município de Rondonópolis (MT), em 2016, foram notificados 102 casos novos, resultando em 45,88/100mil habitantes, sendo o município e o estado caracterizados como regiões hiperendêmicas para a doença.^( 3 , 4 )^

Segundo a Organização Mundial da Saúde (OMS), a qualidade de vida (QV) é definida como “a percepção do indivíduo sobre a sua posição na vida, no contexto da cultura e dos sistemas de valor nos quais ele vive e em relação a seus objetivos, expectativas, padrões e preocupações”.^( 5 )^Sua compreensão perpassou a concepção de sinônimo de saúde e bem-estar, tendo envolvimento com saúde, educação, transporte, moradia, trabalho e participação nas decisões que lhes dizem respeito.^( 6 )^

A hanseníase é capaz de impactar negativamente na QV dos indivíduos. Em geral, a doença traz muito desconforto físico e dores pelo corpo, impedindo o indivíduo de trabalhar e realizar tarefas diárias, impondo limitações às suas atividades e diminuição das atividades de lazer.^( 7 )^

O preconceito e os estigmas impostos pela sociedade, devido às alterações dermatológicas e às deficiências físicas, contribuem para o surgimento de sentimentos, como baixa autoestima, vergonha, rejeição e isolamento no meio familiar, social, acadêmico e profissional. Além desses fatores, o tratamento prolongado e seus efeitos adversos também contribuem negativamente para a QV dos pacientes com hanseníase.^( 2 , 8 )^

A QV no campo da saúde tem um valor atribuído à vida com base nas deteriorações funcionais, percepções e condições sociais induzidas por doenças, agravos e tratamentos, e seu estudo e avaliação têm intuito de humanizar a assistência.^( 9 )^Nesse contexto, uma melhor compreensão acerca da QV dos indivíduos com hanseníase pode contribuir para a ampliação do cuidado de forma integral, bem como no desenvolvimento de intervenções que possam proporcionar melhoria no serviço prestado a essa população.

## OBJETIVO

Avaliar a qualidade de vida e seus fatores associados em pacientes com hanseníase.

## MÉTODOS

Trata-se de estudo descritivo de delineamento transversal e abordagem quantitativa. Foram incluídos na pesquisa todos os pacientes com diagnóstico de hanseníase, com idade igual ou superior a 18 anos, que compareceram ao Serviço de Atendimento Especializado (SAE) Abel Christian Rodrigues de Melo, da cidade de Rondonópolis (MT), no mês de setembro de 2019. Houve três recusas, totalizando 63 pacientes.

Os dados sociodemográficos foram coletados por meio de um questionário estruturado, previamente testado. Foi utilizado o questionário *World Health Organization Quality of Life Bref* (WHOQOL-BREF)^( 10 )^em sua versão em português, disponível em https://www.ufrgs.br/qualidep/qualidade-de-vida/projeto-whoqol-bref/50-whoqol-bref. Esse questionário é validado pela OMS e composto por 26 itens, sendo dois de autoavaliação de saúde e 24 referentes a quatro domínios: físico, psicológico, relações sociais e meio ambiente.

A variável dependente foi QV, e as variáveis independentes foram idade, sexo, cor autodeclarada, escolaridade, renda, situação conjugal, casa própria, número de cômodos da casa, número de pessoas que moram na mesma casa, filiação a plano de saúde, saneamento básico, utilização dos serviços da Estratégia Saúde da Família (ESF) e visita domiciliária de Agentes Comunitários de Saúde (ACS).

Os dados referentes ao questionário WHOQOL-BREF foram analisados inicialmente no programa Excel, conforme descrito por Pedroso et al., utilizando a plataforma disponível em http://www.brunopedroso.com.br/whoqol-bref.html.^( 11 )^Nas questões do WHOQOL-BREF, as médias mais altas sugerem melhor percepção de QV.

As variáveis que apresentaram p<0,20 na análise univariada foram inseridas no modelo de regressão linear múltipla. O nível de significância adotado foi de p≤0,05. Para as análises estatísticas, foram utilizados os programas Excel do Office 365 e o IBM (SPSS) 26.0 for Windows.

Durante a pesquisa, foram seguidos os preceitos éticos de acordo com a Resolução 466, de 12 de dezembro de 2012, do Conselho Nacional de Saúde do Ministério da Saúde, para pesquisa com seres humanos.^( 12 )^Este estudo fez parte da pesquisa intitulada Hanseníase: Análise dos Casos e da Gestão do Programa em um Município Hiperendêmico e foi aprovado pelo Comitê de Ética em Pesquisa da Universidade Federal de Rondonópolis (UFR), parecer 3.036.673 e CAAE: 97441618.2.0000.8088. Todos os participantes do estudo assinaram o Termo de Consentimento Livre Esclarecido (TCLE).

## RESULTADOS

A média de idade dos pesquisados foi de 51,74 anos. A maioria dos participantes era homem (63,50%; n=40), com idade de 30 a 50 anos (44,40%; n=28), autodeclarou-se de cor parda (58,70%; n=37), tinha companheiro (62,90%; n=39), possuía renda maior que dois salários mínimos (54,0%, n=34), estudou até 8 anos (69,80%; n=44), possuía rede de esgoto (60,30%; n=38), não possuía casa própria (75,80%; n=47), tinha mais que quatro cômodos na casa (43,50%; n=27), três ou mais pessoas moravam em sua residência (55,60%; n=35), recebia visita domiciliária do ACS (60,30%; n=38), utilizava a ESF (84,10%; n=53) e não era filiada a plano de saúde (81,0%; n=51).

A figura 1 apresenta as médias dos escores dos domínios de QV do WHOQOL-BREF da população estudada. A pontuação média do WHOQOL-BREF na QV geral foi 14,80±1,89. A maior média foi observada no domínio psicológico (16,28±2,30) e a menor, no domínio ambiente (13,86±2,21).

**Figura 1:** Medidas de centralidade e dispersão dos escores dos domínios de qualidade de vida do *World Health Organization Quality of Life Bref* de indivíduos com hanseníase acompanhados em um serviço de referência

QV: qualidade de vida.

As mulheres (p=0,036), os indivíduos sem companheiro (p=0,016) e aqueles que possuíam casa própria (p=0,033) demonstraram menores valores de QV no domínio psicológico ( Tabelas 1 e 2 ).

**Tabela 1:** Distribuição dos escores médios da qualidade de vida geral e em cada domínio do *World Health Organization Quality of Life Bref* para os pacientes com hanseníase atendidos por um serviço de referência, de acordo com suas características intraindividuais

Variáveis	Média (DP) dos escores WHOQOL-BREF				

	Físico	Psicológico	Relações sociais	Ambiente	QV geral
Sexo					
Feminino	13,46 (2,90)	15,48 (2,65)	15,83 (2,25)	13,39 (2,12)	14,28 (1,95)
Masculino	14,43 (2,76)	16,73 (1,97)	15,53 (2,73)	14,13 (2,23)	15,09 (1,82)
Valor de p	0,196	0,036	0,664	0,202	0,101
Idade em anos					
30-50	14,18 (3,26)	16,09 (2,19)	15,76 (2,46)	13,52 (2,23)	14,71 (2,02)
51-59	13,53 (2,86)	16,24 (2,79)	15,44 (2,68)	14,42 (2,53)	14,80 (2,18)
> 60	14,53 (1,85)	16,62 (1,94)	15,66 (2,69)	13,80 (1,68)	14,94 (1,31)
Valor de p	0,567	0,768	0,914	0,390	0,932
Cor autodeclarada					
Branca	13,75 (3,39)	16,27 (3,18)	15,37 (3,23)	14,49 (2,26)	14,83 (2,26)
Parda	14,35 (2,47)	16,16 (2,01)	15,75 (2,35)	13,94 (2,09)	14,92 (1,73)
Preta	13,59 (3,25)	16,74 (1,50)	15,70 (2,08)	12,33 (2,09)	14,24 (1,88)
Valor de p	0,066	0,801	0,882	0,054	0,633
Situação conjugal					
Sem companheiro	13,79 (2,74)	15,39 (2,50)	15,13 (2,62)	13,67 (2,16)	14,41 (1,85)
Com companheiro	14,39 (2,78)	16,84 (2,03)	16,00 (2,48)	14,08 (2,16)	15,12 (1,81)
Valor de p	0,413	0,016	0,197	0,473	0,142

DP: desvio padrão; WHOQOL-BREF: *World Health Organization Quality of Life Bref;* QV: qualidade de vida.

**Tabela 2:** Distribuição dos escores médios da qualidade de vida geral e em cada domínio do *World Health Organization Quality of Life Bref* para os pacientes com hanseníase atendidos por um serviço de referência, de acordo com suas características socioeconômicas

Variáveis	Média (DP) dos escores WHOQOL-BREF				

	Físico	Psicológico	Relações sociais	Ambiente	QV geral
Escolaridade					
Até 8 anos	13,71 (3,00)	16,34 (2,33)	15,70 (2,65)	13,69 (2,39)	14,66 (2,06)
Mais de 8 anos	14,92 (2,24)	16,10 (2,28)	15,51 (2,36)	14,26 (1,68)	15,12 (1,43)
Valor de p	0,122	0,704	0,790	0,347	0,383
Renda					
< 1 salário mínimo	13,08 (3,17)	15,70 (2,55)	15,17 (2,65)	13,29 (2,35)	14,14 (2,10)
> 2 salários mínimos	14,92 (2,20)	16,76 (1,97)	16,04 (2,42)	14,34 (1,98)	15,35 (1,52)
Valor de p	0,009	0,067	0,180	0,058	0,011
Casa própria					
Sim	13,40 (3,23)	15,20 (1,97)	15,47 (2,56)	13,23 (2,49)	14,19 (1,97)
Não	14,40 (2,58)	16,65 (2,32)	15,74 (2,57)	14,15 (2,01)	15,07 (1,77)
Valor de p	0,226	0,033	0,717	0,150	0,111
Número cômodos					
1-3	12,95 (2,87)	14,81 (3,43)	15,11 (3,26)	13,50 (2,16)	13,91 (2,05)
4	14,72 (2,43)	16,82 (1,69)	16,46 (1,69)	14,26 (2,47)	15,35 (1,88)
> 5	14,03 (2,97)	16,30 (2,68)	15,11 (2,09)	13,76 (1,83)	14,70 (1,65)
Valor de p	0,241	0,079	0,120	0,572	0,112
Número de pessoas na residência					
< 2	13,73 (2,40)	15,73 (1,69)	15,46 (2,85)	13,68 (2,32)	14,50 (1,60)
> 3	14,50 (3,00)	16,74 (2,63)	15,85 (2,33)	14,13 (2,02)	15,13 (1,99)
Valor de p	0,285	0,087	0,554	0,417	0,189
Rede de esgoto					
Sim	14,39 (2,56)	16,40 (2,34)	15,61 (2,50)	13,99 (1,92)	14,92 (1,68)
Não	13,60 (3,18)	16,08 (2,28)	15,68 (2,28)	15,68 (2,67)	14,61 (2,20)
Valor de p	0,281	0,589	0,921	0,583	0,522

DP: desvio padrão; WHOQOL-BREF: *World Health Organization Quality of Life Bref* ; QV: qualidade de vida.

Os indivíduos com menor renda apresentaram menor média nos escores de QV geral (p=0,011) e no domínio físico (p=0,009) ( Tabela 2 ).

Os pacientes que não recebem visita de ACS evidenciaram pior QV no domínio ambiente (p=0,040) ( Tabela 3 ).

**Tabela 3:** Distribuição dos escores médios da qualidade de vida geral e em cada domínio do *World Health Organization Quality of Life Bref* para os pacientes com hanseníase atendidos por um serviço de referência, de acordo com suas características de acesso à saúde

Variáveis	Média (DP) dos escores WHOQOL-BREF				

	Físico	Psicológico	Relações sociais	Ambiente	QV geral
Utiliza ESF					
Sim	13,98 (2,71)	16,26 (2,21)	15,90 (2,52)	13,80 (2,22)	14,78 (1,84)
Não	14,57 (3,50)	16,33 (2,85)	14,27 (2,35)	14,20 (2,21)	14,91 (2,24)
Valor de p	0,551	0,931	0,063	0,601	0,843
Recebe visita de ACS					
Sim	13,95 (3,07)	16,31 (2,43)	15,86 (2,46)	14,32 (2,23)	14,95 (2,03)
Não	14,26 (2,46)	16,21 (2,12)	15,31 (2,70)	13,16 (2,01)	14,57 (1,68)
Valor de p	0,676	0,864	0,404	0,040	0,448
Plano de saúde					
Sim	13,24 (2,78)	15,94 (2,40)	15,66 (2,28)	13,79 (2,05)	14,42 (1,59)
Não	14,27 (2,83)	16,35 (2,29)	15,63 (2,63)	13,88 (2,26)	14,89 (1,96)
Valor de p	0,257	0,584	0,969	0,904	0,451

DP: desvio padrão; WHOQOL-BREF: *World Health Organization Quality of Life Bref* ; QV: qualidade de vida; ESF: Estratégia Saúde da Família; ACS: Agente Comunitário de Saúde.

Os resultados da regressão multivariada estão representados na tabela 4 . Após a análise ajustada, as variáveis que permaneceram associadas à QV foram escolaridade e cor autodeclarada. Menor escolaridade foi associada à menor QV em relação ao domínio físico, e ser branco foi associado à uma melhor QV no domínio ambiente.

**Tabela 4:** Análise multivariada dos escores médios da qualidade de vida geral e em cada domínio do *World Health Organization Quality of Life Bref* para os pacientes com hanseníase atendidos por um serviço de referência

Variáveis	Coeficiente de correlação				

	Físico (IC95%)	Psicológico (IC95%)	Relações sociais (IC95%)	Ambiente (IC95%)	QV geral (IC95%)
Sexo	-0,50 (-2,16 - -1,17)	-0,27 (-1,60-1,06)	*	*	-0,27 (-1,51-0,96)
Cor autodeclarada	*	*	*	1,39^†^(0,28-2,50)	*
Escolaridade	-1,78^†^(-3,51 - -0,04)	*	*	*	*
Renda	*	-0,016 (-0,67-0,64)	0,39 (-0,30-1,08)	-0,18 (-0,85-0,49)	0,08 (-0,52-0,67)
Situação conjugal	*	-0,40 (-1,72-0,92)	*	*	0,10 (-1,23-1,03)
Casa própria	*	0,98 (-0,47-2,43)	*	0,53 (-1,12-2,19)	0,35 (-0,90-1,60)
Número de cômodos	*	-0,313 (-1,55-0,93)	0,63 (-0,71-1,97)	*	-0,06 (-1,13-1,01)
Número de pessoas na residência	*	-0,363 (-1,63-0,90)	*	*	-0,59 (-1,67-0,50)
Utiliza ESF	*	*	0,27 (-1,59-2,13)	*	*
Recebe visita do ACS	*	*	*	0,61 (-0,75-1,97)	*

* p>0,20 na análise univariada, não foram incluídos na regressão logística. Análise multivariada;^†^p≤0,05.

IC95%: intervalo de confiança de 95%; QV: qualidade de vida; ESF: Estratégia Saúde da Família; ACS: Agente Comunitário de Saúde.

## DISCUSSÃO

A hanseníase como doença que vem atingindo intensas proporções no mundo todo pode afetar o indivíduo em diversas dimensões e trazer impactos sobre sua QV.

Neste estudo, a maioria dos participantes era de homens, autodeclarou-se de cor parda, residia com companheiro, possuía renda familiar maior ou igual a dois salários mínimos e referia até 8 anos de estudo. Esses dados corroboram pesquisas anteriores, que indicam que homens, pessoas com condições socioeconômicas precárias, aqueles com baixo nível de instrução e indivíduos com baixa renda apresentam maior risco de adquirir a doença.^( 13 - 16 )^

Evidenciamos, na presente pesquisa, que o domínio do WHOQOL-BREF com a menor média foi o domínio ambiente. Esse domínio refere-se às facetas de proteção, como disponibilidade e qualidade dos cuidados de saúde e sociais, oportunidade de adquirir novas informações e habilidades.^( 17 )^Esses fatores podem interferir no processo de autocuidado e no sucesso terapêutico da hanseníase. Nesse sentido, é importante que os parâmetros ambientais sejam levados em consideração para adoção das estratégias de promoção da saúde nesse grupo populacional.

De modo semelhante, pesquisa com pacientes com hanseníase acompanhados em centros de referência especializados em Sergipe (AL) também descreve que o domínio ambiente apresentou os menores escores no WHOQOL-BREF.^( 18 )^Um estudo realizado com pacientes de grupos de apoio ao autocuidado em hanseníase de Recife (PE) e Região Metropolitana verificou menores escores de QV para os domínio ambiente e físico, ambos com mesma média.^( 19 )^No entanto, estudos prévios observaram que o domínio físico apresentou as menores médias de QV em populações de indivíduos com hanseníase.^( 16 , 20 )^

Apesar do WHOQOL-BREF permitir a comparação de seus resultados entre diferentes populações, há de se levar em consideração que a noção de QV sofre influências pessoais relacionadas à capacidade individual de lidar com determinadas situações. Esse fato, somado a fatores ambientais, sociais e culturais locais, poderá influenciar nos resultados do teste, quando realizado em diferentes localidades.^( 21 )^

Na presente pesquisa, os fatores que se mantiveram associados à QV na regressão múltipla foram cor autodeclarada e escolaridade. Brouwers et al., demonstraram que manter uma família, satisfação com a saúde, limitações para realização das atividades, e tipo de casa foram fatores significativamente relacionados à uma pior QV em pacientes com hanseníase do Nepal.^( 22 )^Tsutsumi et al., verificaram que os fatores que potencialmente contribuem para pior QV em pacientes com hanseníase foram a presença de estigma percebido, menos anos de estudo, presença de deformidades e uma renda anual menor.^( 16 )^

Após análise multivariada, verificou-se que a melhor QV esteve associada à autodeclaração de cor branca no domínio ambiente. Dados do Instituto Brasileiro de Geografia e Estatística (IBGE) indicam que a população preta ou parda se apresenta em desvantagem em questões como mercado de trabalho, rendimento médio mensal, saneamento básico, acesso à Internet, anos de estudo e segurança pública.^( 23 )^Essas questões são englobadas no domínio ambiente, podendo justificar o fato de os pacientes brancos apresentarem melhor QV. Esse resultado reforça a necessidade de adoção de políticas públicas que busquem reduzir as desigualdades sociais, garantindo melhores condições de vida a todas às parcelas da população.

Menor escolaridade foi um fator associado negativamente à QV no domínio físico, e resultado semelhante foi verificado em pacientes com hanseníase atendidos em um hospital de Bangladesh.^( 16 )^Leite et al., analisaram a QV de indivíduos com hanseníase nos municípios de Bayeux (PA) e de Natal (RN). Os autores verificaram que as menores médias de QV total e do domínio de relações sociais foram observadas em indivíduos com menos estudo.^( 20 )^

O domínio físico do WHOQOL-BREF visa avaliar até que ponto energia e fadiga, dor e desconforto, sono e repouso são afetados.^( 16 )^Uma baixa escolaridade pode acarretar dificuldades no entendimento das orientações realizadas pela ESF, assim como no seguimento terapêutico.^( 21 )^Nesse contexto, os indivíduos com menor escolaridade poderão apresentar mais dificuldades de autocuidado, o que poderá impactar em sua QV. Esse dado é um importante indicador a ser observado pelos gestores e profissionais no planejamento das ações de saúde, de modo a se refinar o olhar e a propor estratégias direcionadas aos pacientes menos escolarizados.^( 21 )^

Atualmente, existem poucos estudos brasileiros que avaliam os fatores associados à QV de pacientes com hanseníase, demonstrando a importância de ampliação de estudos que abordem esse tema. Quanto às limitações deste estudo, pode-se citar o método de coleta de dados, em que as informações são autorrelatadas, tanto no que se refere aos dados sociodemográficos quanto ao WHOQOL-BREF, o que pode estar sujeito a viés de memória. O WHOQOL-BREF, porém, é um instrumento validado e amplamente utilizado. Outra limitação, refere-se a amostra estuda, que envolveu um pequeno grupo de pacientes acompanhados por um serviço de referência, sem uma amostragem dos pacientes do município. Devem ser realizados estudos futuros mais abrangentes.

## CONCLUSÃO

Indivíduos com hanseníase acompanhados por um serviço de referência apresentaram baixos escores de qualidade de vida. As percepções de pior qualidade de vida foram observadas entre as mulheres, os indivíduos de baixa escolaridade, da cor parda ou preta, sem companheiro, que não possuíam casa própria e que não recebiam visita de Agentes Comunitários de Saúde. Os resultados indicaram o impacto dos fatores sociodemográficos sobre a qualidade de vida, reforçando a necessidade de realização de uma assistência em saúde de maneira integral, compreendendo os determinantes sociais da saúde dessa população.
